# Soluble P-selectin rescues viper venom–induced mortality through anti-inflammatory properties and PSGL-1 pathway-mediated correction of hemostasis

**DOI:** 10.1038/srep35868

**Published:** 2016-10-25

**Authors:** Der-Shan Sun, Pei-Hsun Ho, Hsin-Hou Chang

**Affiliations:** 1 Department of Molecular Biology and Human Genetics, Tzu-Chi University, Hualien 970, Taiwan; 2 Center for Vascular Medicine, Tzu-Chi University, Hualien 970, Taiwan

## Abstract

Venomous snakebites are lethal and occur frequently worldwide each year, and receiving the antivenom antibody is currently the most effective treatment. However, the specific antivenom might be unavailable in remote areas. Snakebites by *Viperidae* usually lead to hemorrhage and mortality if untreated. In the present study, challenges of rattlesnake (*Crotalus atrox*) venom markedly increased the circulating soluble P-selectin (sP-sel) level, but not P-selectin (P-sel, *Selp*^−/−^) mutants, in wild-type mice. Because sP-sel enhances coagulation through the P-selectin ligand 1 (PSGL-1, *Selplg*) pathway to produce tissue factor–positive microparticles, we hypothesized that increasing the plasma sP-sel level can be a self-rescue response in hosts against snake venom–mediated suppression of the coagulation system. Confirming our hypothesis, our results indicated that compared with wild-type mice, *Selp*^−/−^ and *Selplg*^−/−^ mice were more sensitive to rattlesnake venom. Additionally, administration of recombinant sP-sel could effectively reduce the mortality rate of mice challenged with venoms from three other *Viperidae* snakes. The antivenom property of sP-sel is associated with improved coagulation activity *in vivo*. Our data suggest that the elevation of endogenous sP-sel level is a self-protective response against venom-suppressed coagulation. The administration of recombinant sP-sel may be developed as a new strategy to treat *Viperidae* snakebites.

Venomous snakebites are a major cause of life-threatening human injury from poisonous animals worldwide^1^. Currently available data suggest that the true effects of snake bites are underestimated^2^. For example, snake bites are a crucial occupational hazard affecting farmers, plantation workers, herders, and fishermen; open-style habitation and the practice of sleeping on floors also expose people to snake bites^2^. *Viperidae*, a flourishing snake family, contains more than 200 species of venomous snakes belonging to two subfamilies: pit vipers (*Crotalinae*) and old-world vipers (*Viperinae*)^3^. These snakes eat small animals and hunt by striking and envenoming to immobilize and kill prey^4^. Their venom comprises various coagulation-disrupting proteins^1^,^5^, which can cause functional alterations in coagulation factors and platelets^5^,^6^,^7^,^8^,^9^,^10^,^11^,^12^,^13^,^14^,^15^,^16^,^17^,^18^,^19^; thus, snakebites by vipers usually lead to inflammation and hemorrhage^1^,^2^,^5^,^20^,^21^,^22^,^23^. Antivenom injection is a major treatment for venomous snakebites, but little high-quality data is available to support its effectiveness, particularly in cases with venom-elicited coagulopathy^1^,^5^,^24^,^25^. Additionally, treatment with antivenom is not risk free, and adverse effects are common and occasionally severe^1^. Furthermore, because snakebites usually occur within a snake habitat, the specific antivenom may not be available in local hospitals^2^. Therefore, an alternative treatment is needed for venom-induced coagulant defect.

P-selectin (P-sel), a cell-surface adhesion molecule, is a member of the selectin family and is expressed and stored in the intracellular vesicle α-granules of platelets and the Weibel–Palade bodies of endothelial cells. P-sel is translocated to cell surfaces after stimulation^26^ and binds to oligosaccharide sialylated Lewis x and heparan sulfate containing glycoproteins, particularly the primary ligand, P-sel ligand 1 (PSGL-1)^27^,^28^. Increase in circulating soluble P-sel (sP-sel) level is associated with thrombotic consumptive disorders such as disseminated intravascular coagulation and thrombotic thrombocytopenic purpura, which involves generalized hypercoagulation^29^. However, the pathophysiological role of sP-sel in these diseases remains unclear. Recombinant sP-sel treatment in mice is associated with induction of the procoagulant state^30^ and can correct hemostasis in a mouse model of hemophilia A through interaction with PSGL-1 to generate procoagulant tissue factor (TF)-positive microparticles (MPs) (TF^+^-MPs)^31^. In this study, we demonstrated that challenges of rattlesnake venom increases circulating sP-sel level in mice. Although the mechanism by which sP-sel induces the procoagulant state remains elusive, the increases in sP-sel and coagulation levels in venom-challenged mice may help the host survive hemorrhage pathogenesis. Therefore, we hypothesized that the increase in circulating sP-sel could be a self-rescue response against venom-induced bleeding. The relevant mechanism and potential applications are discussed.

## Results

### Mortality and abnormal coagulant pathogenesis induced by snake venom of *Crotalus atrox*

**Figure 1:**
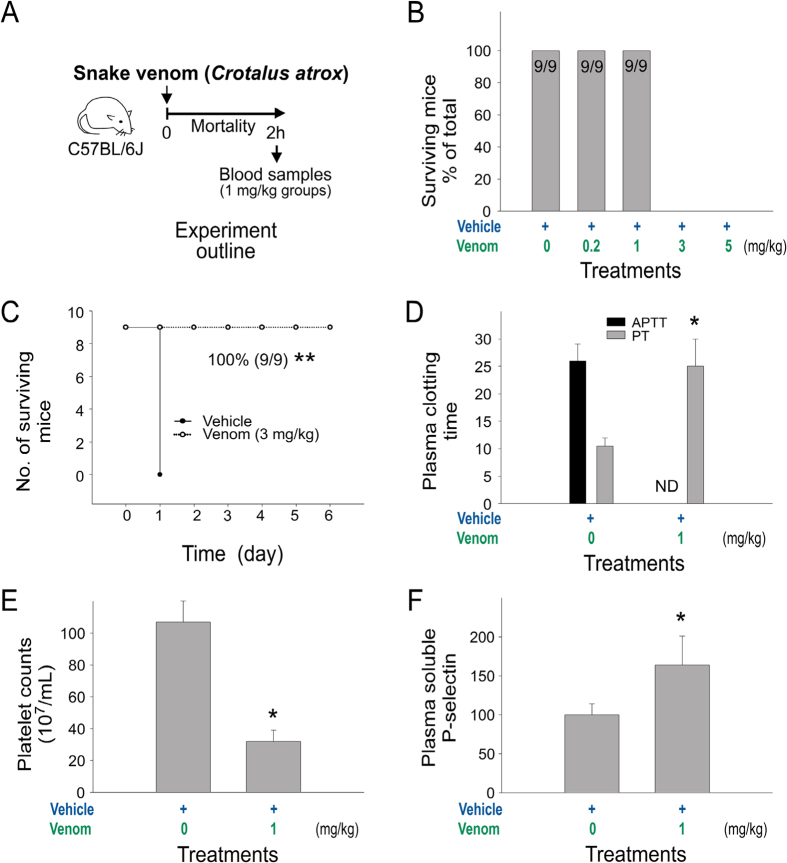
Mortality and abnormal coagulant pathogenesis induced by snake venom of *Crotalus atrox*, the western diamondback rattlesnake. (**A**) Experiment outline; (**B**) survival rates of C57BL/6J mice challenged with different dosages of the venom (n = 9); (**C**) mortality of mice challenged with 3 mg/mL venom plotted as Kaplan–Meier curves (n = 9); (**D**) plasma-clotting time analyses (APTT: activated partial thromboplastin time; PT: prothrombin time) (ND: no detectable clotting); (**E**) indicated platelet counts and (**F**) plasma soluble P-selectin (sP-sel) (n = 6) (**D–F**); 3 independent experiments with 2 or 3 replicates). Control Ig vs. rP-sel-Fc, **P = 3.7 × 10^−5^ (**C**); vehicle vs. venom,*P < 0.05 (**D**–**F**). The mouse drawing used in this figure was originally published in the Blood journal: Huang, H. S., Sun, D. S., Lien, T. S. and Chang, H. H. Dendritic cells modulate platelet activity in IVIg-mediated amelioration of ITP in mice. Blood, 2010; 116: 5002–5009^58^. © the American Society of Hematology.

The rattlesnake (*Crotalus atrox*) venom–induced mortality in wild-type C57BL/6J mice was examined. An injection dose higher than 3 mg/kg was found to cause 100% mortality, whereas doses lower than 1 mg/kg were not lethal (Fig. 1A experiment outline; 1B, mortality; 1C, Kaplan–Meier survival curves). In mice injected with sublethal doses of venom (1 mg/kg), we found prolonged plasma clotting times, reduced platelet counts, and increased plasma sP-sel level, all of which indicated that the coagulation system was suppressed (Fig. 1D–F).

### P-selectin and P-selectin ligand 1 mutants are more sensitive to venom challenges than the wild type mice

**Figure 2:**
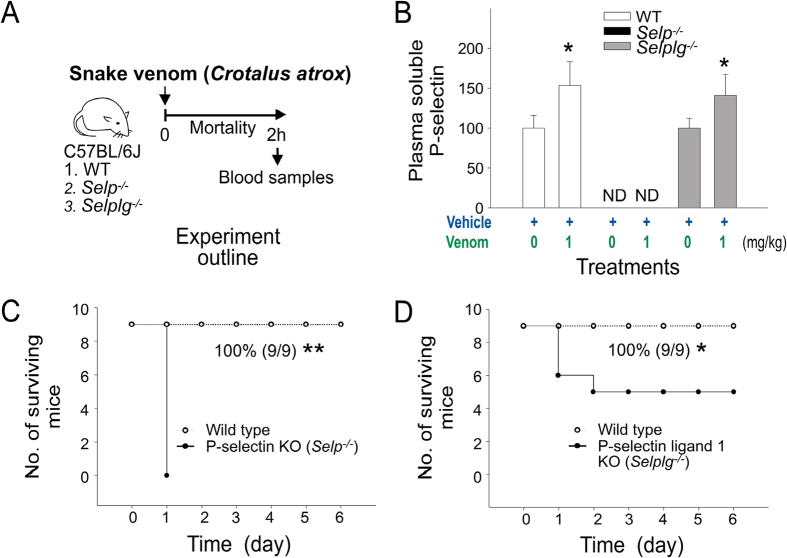
Soluble P-selectin (sP-sel) levels and mortality in venom-treated mice. (**A**) Experiment outline, *Crotalus atrox* venom (1 mg/kg) elicited; (**B**) sP-sel levels (% of respective vehicle controls); (**C**) mortality in wild-type (C57BL/6J; *Selp*^+/+^, *Selplg*^+/+^) vs. P-sel KO (C57BL/6J; *Selp*^−/−^); (**D**) wild-type vs. PSGL-1 KO (C57BL/6J; *Selplg*^−/−^). *P < 0.05, venom vs. respective vehicle groups, n = 9 (**B**). ND: none detectable. Mortality is plotted as Kaplan–Meier curves [WT vs. KO, **P = 3.7 × 10^−5^ (**C**); *P = 2.7 × 10^−2^ (**D**), n = 9]. The mouse drawing used in this figure was originally published in the Blood journal: Huang, H. S., Sun, D. S., Lien, T. S. and Chang, H. H. Dendritic cells modulate platelet activity in IVIg-mediated amelioration of ITP in mice. Blood, 2010; 116: 5002–5009^58^. © the American Society of Hematology.

Injections of sP-sel are known to induce a procoagulant state through interaction with P-selectin ligand 1 (PSGL-1), which leads to the production of tissue factor–positive MPs (TF^+^-MPs) that are beneficial for a suppressed coagulation system in the hemophilia A mouse model^31^. Therefore, we hypothesized that the elevation of circulating sP-sel could be a native self-rescue response to counteract the suppressed coagulation system. Thus, compared with wild-type mice, P-sel knockout (KO) mice should be more sensitive to venom challenges because they do not produce sP-sel. Similarly, because sP-sel requires functional PSGL-1 to cause an increase in TF^+^-MP counts, PSGL-1 KO mice should be more sensitive to venom challenges compared with the parental strain. In the present study, we used sublethal doses of snake venom for wild-type mice to treat P-sel and PSGL-1 KO mice. In agreement with our hypothesis, we found that a sublethal dose of snake venom (1 mg/kg; Fig. 1B) markedly increased soluble P-sel in the wild-type and PSGL-1 (*Selplg*^−/−^) KO mice, but not in P-sel (*Selp*^−/−^) KO mice (Fig. 2A experiment outline, and 2B). In addition, a sublethal dose for wild-type mice (1 mg/kg; Fig. 1B) was lethal in P-sel KO mice and PSGL-1 KO mice (Fig. 2C,D). These results suggest that P-sel and PSGL-1 are vital for mice to counteract snake venom–mediated toxicity.

### Injection of recombinant sP-sel-IgG Fc fusion protein rescues venom-challenged mice

**Figure 3:**
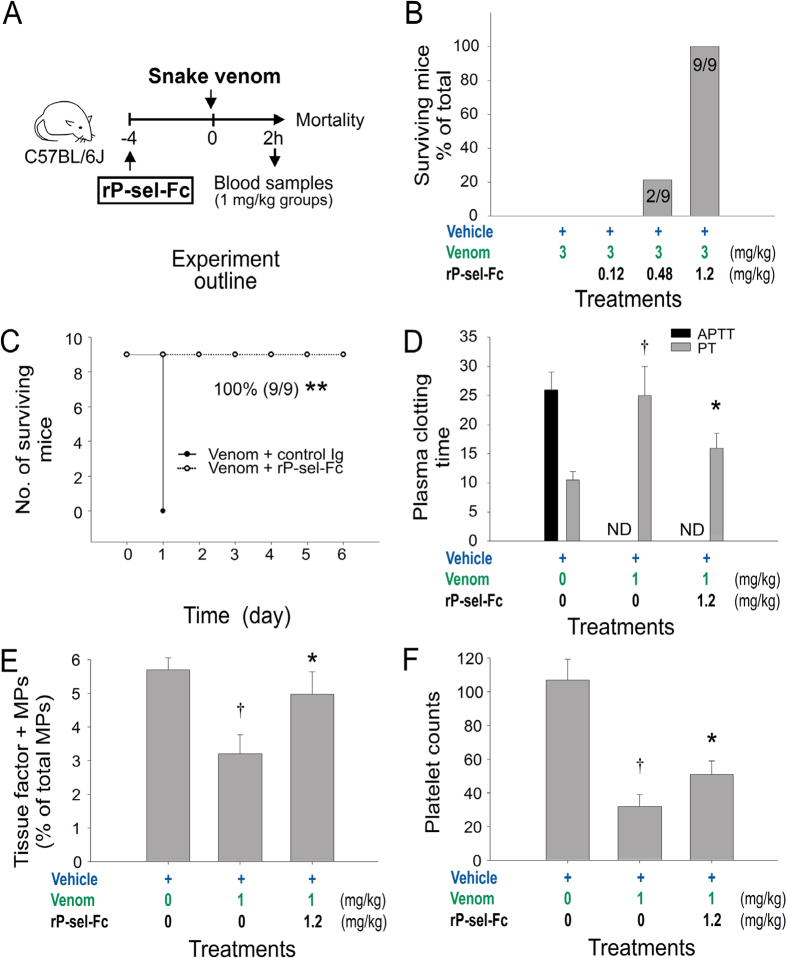
Recombinant sP-sel-IgG Fc fusion protein (rP-sel-Fc)-mediated rescue in venom-challenged mice. (**A**) Experiment outline; (**B**,**C**) rP-sel-Fc-rescued mortality (n = 9); (**D**) plasma-clotting time APTT and PT; (**E**) indicated tissue factor-positive microparticles (TF^+^-MPs) of the venom-treated mice. ND: no detectable clotting (n = 6) (**D**–**F)**; 3 independent experiments with 2 or 3 replicates). **P = 3.7 × 10^−5^, control Ig vs. rP-sel-Fc (**C**), ^†^*P* < 0.05, vehicle vs. venom, *P < 0.05, venom vs. venom + rP-sel-Fc groups (**D**–**F**). The mouse drawing used in this figure was originally published in the Blood journal: Huang, H. S., Sun, D. S., Lien, T. S. and Chang, H. H. Dendritic cells modulate platelet activity in IVIg-mediated amelioration of ITP in mice. Blood, 2010; 116: 5002–5009^58^. © the American Society of Hematology.

To further determine whether injections of sP-sel were beneficial to the mice during venom challenges, the rescue effect of the injection of recombinant sP-sel in venom-challenged mice was investigated. Our data indicated that recombinant P-sel IgG-Fc fusion protein (rP-sel-Fc) exerted a protective effect against venom-induced mortality in mice (Fig. 3A experiment outline, 3B,C). In addition, treatments of rP-sel-Fc markedly improved the coagulant parameters, including suppressed TF^+^-MPs levels and prolonged plasma clotting time and thrombocytopenia in venom-challenged mice (1 mg/kg, a sublethal dose; Fig. 3D–F).

### Anti-P-sel neutralizing Ig-mediated rescue

**Figure 4:**
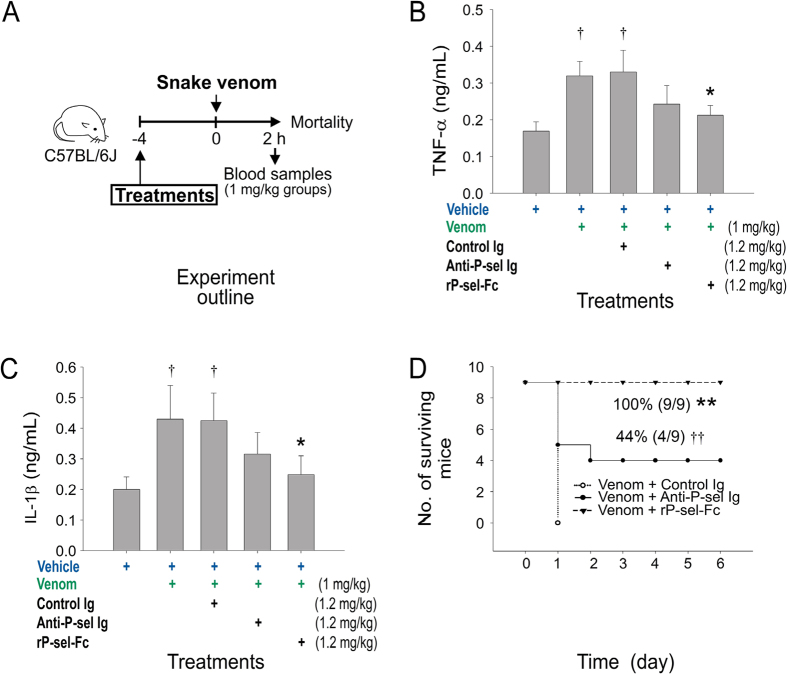
Anti-P-sel neutralizing Ig (anti-P-sel Ig)-mediated rescue. (**A**) Experiment outline; (**B**,**C**) anti-P-sel Ig-mediated reduction of mouse circulating TNF-α and IL-1β (n = 6); (**D**) indicated mortality (n = 9) of venom-treated mice. ^†^*P* < 0.05, vs. vehicle groups, *P < 0.05, vs. control Ig groups (**B**,**C**). **P = 3.7 × 10^−5^, ^††^*P* = 1 × 10^−2^, vs. control Ig groups (**D**). The mouse drawing used in this figure was originally published in the Blood journal: Huang, H. S., Sun, D. S., Lien, T. S. and Chang, H. H. Dendritic cells modulate platelet activity in IVIg-mediated amelioration of ITP in mice. Blood, 2010; 116: 5002–5009^58^. © the American Society of Hematology.

Because PSGL-1 KO mice are less sensitive than the P-sel KO mice (Fig. 2C vs. 2D), and because PSGL-1 is essential for the induction of TF^+^-MPs in eliciting a procoagulant response^31^, our results suggest that additional protective mechanisms may be involved. Because P-sel is an adhesion molecule on platelets and endothelial cells that mediates the interaction of these cells with leukocytes, recombinant sP-sel and P-sel-neutralizing antibodies can block leukocyte infiltration to the inflamed tissues and exert an anti-inflammatory effect^27^. Thus, in addition to the coagulant-modulation effect, the anti-inflammatory effect of sP-sel may contribute to the protection against venom challenges. To investigate this possibility, we employed a P-sel-neutralizing antibody. We found that, although not fully protected, injections of the P-sel neutralizing antibody tended to reduce the circulating levels of proinflammatory cytokines TNF-α and IL-1β, and markedly reduced the mortality rate of venom-challenged mice (Fig. 4A experiment outline, 4B–D). These results suggest that the anti-inflammatory effect is also involved in sP-sel-mediated amelioration of snake venom–induced mortality in mice.

### The rP-sel-Fc mediated rescue of challenges with different viper venoms in mice

**Figure 5:**
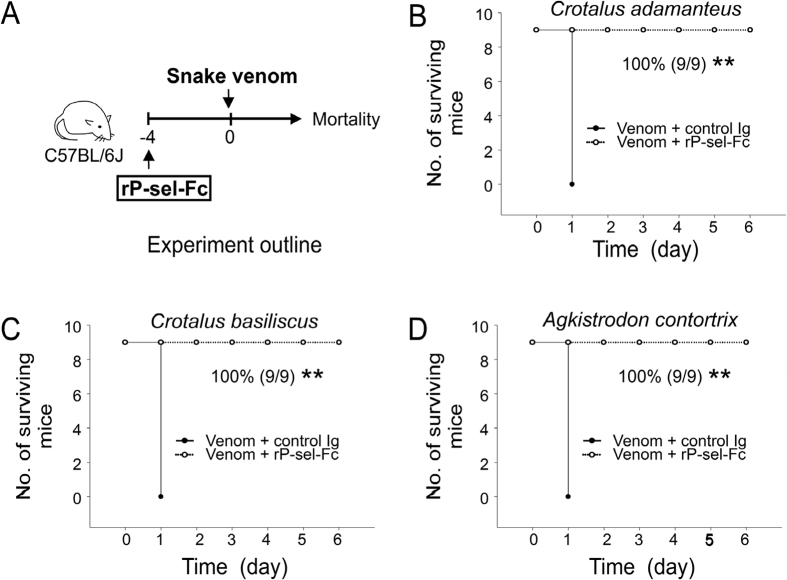
rP-sel-Fc-mediated rescue of viper venom-induced mortality in mice. (**A**) Experiment outline and indicated lethal dose injections using venoms from (**B**) *Crotalus adamanteus* (eastern diamondback rattlesnake), (**C**) *Crotalus basiliscus* (mexican west-coast rattlesnake), and (**D**) *Agkistrodon contortrix* (copperhead) in the mouse model. Mortality is plotted as Kaplan–Meier curves (control Ig vs. rP-sel-Fc, **P = 3.7 × 10^−5^, n = 9) (**B**–**D**). The mouse drawing used in this figure was originally published in the Blood journal: Huang, H. S., Sun, D. S., Lien, T. S. and Chang, H. H. Dendritic cells modulate platelet activity in IVIg-mediated amelioration of ITP in mice. Blood, 2010; 116: 5002–5009^58^. © the American Society of Hematology.

We hypothesized that elevation of circulating sP-sel is a native response to overcome venom-induced inflammation and coagulopathy. Therefore, rP-sel-Fc injection–mediated rescue could be applied to rescue pathogenesis induced by venom of other vipers. To test this hypothesis, mice were challenged with venoms from two rattlesnakes, *Crotalus adamanteus* and *Crotalus basiliscus*, and a viper, *Agkistrodon contortrix*. Mortality analysis indicated that treatments of sP-sel markedly rescued the lethal injections of aforementioned three snake venoms in mice (Fig. 5A experiment outline, 5B–D).

Our results showed that snake venom injections increased circulating sP-sel level and that this increase had a beneficial effect in mice during snake venom challenges.

## Discussion

Blood circulation plays a vital role in the survival of vertebrates, including humans. Problems in blood circulation could be lethal. Hemostasis is the first line of defense against bleeding. Hemostasis involves both clot formation (coagulation) and clot dissolution (fibrinolysis), opposite cooperative processes that keep the hemodynamic properties of the blood constant^32^. The venoms of *Viperidae* snakes contain proteins that can be classified as coagulants, anticoagulants, and fibrinolytic factors^19^. Snakebites usually result in persistent bleeding because the venoms cause considerable degradation of fibrinogen and involve other coagulant factors, which affect platelet function, thus preventing clot formation^19^,^33^. However, whether an emergency assistance system exists *in vivo* to rescue such abnormal coagulation status remains unknown.

Increased levels of circulating sP-sel is observed in various thrombotic consumptive disorders such as heparin-induced thrombocytopenia and haemolytic uremic syndrome, which involve the induction of a procoagulant status^29^. Increased sP-sel level has also been observed in coagulation disorders, infectious diseases, and even tumors^34^. Consequently, an increased plasma sP-sel level has been considered a disease marker in abnormalities involving vascular damage, *in vivo* platelet activation, and thrombosis^34^,^35^,^36^. By contrast, the physiological roles of sP-sel in these diseases remain unclear and have rarely been reported.

Treatments with exogenous rP-sel-Fc have been demonstrated to correct the hemostasis of mice with hemophilia A through interaction with PSGL-1 to elicit procoagulant TF^+^-MPs^31^. If rP-sel-Fc treatments are beneficial for a hemorrhage-prone condition, the elevation of endogenous circulating sP-sel may be a protective response against bleeding. Moreover, increased sP-sel level has been observed in other hemorrhagic diseases such as dengue hemorrhagic fever^37^, immune thrombocytopenia^38^,^39^, and subarachnoid hemorrhage^40^. Despite the findings, the role of sP-sel in these disorders remains unclear. Hemorrhage is a major manifestation of venomous snakebites^5^,^19^. The hemorrhagic pathogeneses are mediated through the suppression of coagulation factors and platelets^19^,^33^. In this study, we report that snake venoms increased plasma sP-sel level in mice. We hypothesized that the increase in sP-sel level is a self-rescue response that led to amelioration of venom-mediated hemorrhage. We found that treatment with recombinant sP-sel markedly ameliorated venom-induced pathogenesis and reduced the mortality rate in mice.

The induction of procoagulation by rP-sel-Fc treatment is mediated through the interaction between rP-sel-Fc and PSGL-1^31^, which suggests that both P-sel and PSGL-1 are essential in counteracting the toxic effects of snake venoms. P-sel and PSGL-1 KO mice are more sensitive to venom challenges compared with wild-type mice. In addition, injections of sP-sel markedly rescued clotting defect and reduced mortality rates in mice. Circulating sP-sel or P-sel neutralizing antibodies also exerted an anti-inflammatory effect by blocking the interaction between endothelial P-sel and leukocyte PSGL-1 to prevent leukocyte infiltration and inflammation in the inflamed tissues^27^. Therefore, we used a sP-sel neutralizing antibody for comparison. We found that the anti-P-sel Ig also exerted a rescuing effect in venom-challenged mice, even though rP-sel-Fc ameliorated venom-stimulated inflammation and mortality more efficiently compared with the anti-P-sel Ig (Fig. 4). These results collectively suggested that both procoagulant (Fig. 3, upregulating TF^+^-MPs) and anti-inflammatory (Fig. 4) properties of sP-sel are involved in the rescue of venom-induced pathogenesis.

**Figure 6:**
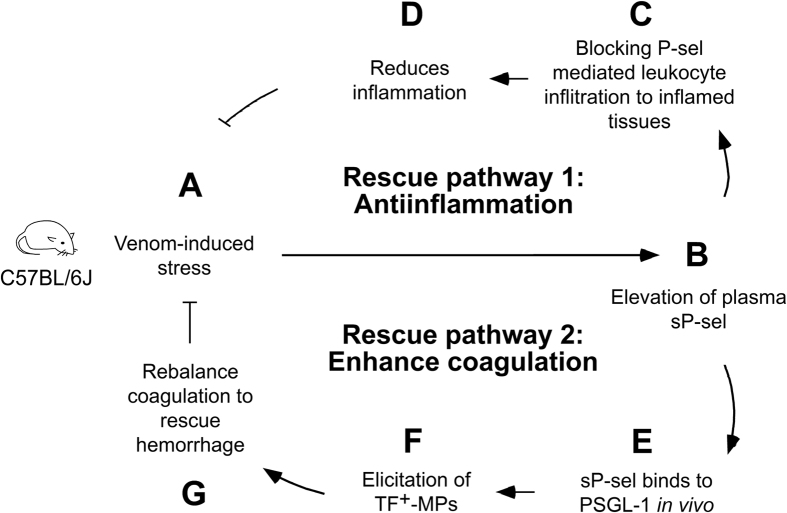
Hypothetical model for sP-sel-mediated rescue of venomous viper snakebites. Data suggest that two rescue pathways are involved. Rescue pathway 1 involves a sP-sel-mediated anti-inflammatory regulation (**A**–**D**). Rescue pathway 2 involves sP-sel and P-sel ligand-1 (PSGL-1)-mediated elicitation of circulating tissue factor-positive microparticles (TF^+^-MPs) for rebalancing the coagulation system (**A**–**B** to **E**–**G**). The mouse drawing used in this figure was originally published in the Blood journal: Huang, H. S., Sun, D. S., Lien, T. S. and Chang, H. H. Dendritic cells modulate platelet activity in IVIg-mediated amelioration of ITP in mice. Blood, 2010; 116: 5002–5009^58^. © the American Society of Hematology.

Here, we propose a model of sP-sel-mediated amelioration of snake venom–induced toxicity (Fig. 6), in which two rescue pathways are involved. In pathway 1, venom-induced stress upregulates circulating sP-sel level (Fig. 6A,B), which exerts anti-inflammatory effects and thus reduces inflammation (Fig. 6C,D). In pathway 2, through a PSGL-1-dependent pathway, sP-sel increases the circulating TF^+^-MP counts to induce a procoagulant state for counteracting venom-mediated suppression of the coagulation system (Fig. 6E–G).

According to this model, however, if the venom induced a hypercoagulable state, how can the procoagulant property of sP-sel still rescue the envenoming host? There are three possible explanations. First, venom-induced hypercoagulation (<2 h; Fig. 1A,D, APTT, PT already prolonged and shifted to hypocoagulation within 2 h; Suppl. Fig. 1C-2, blue labels) and sP-sel-elicited procoagulation (>6 h; Suppl. Fig. 2) do not appear simultaneously. The anti-inflammatory and procoagulant properties of sP-sel are elicited according to chronological order. We found that rP-sel-Fc exerted its anti-inflammatory property relatively quickly, within one hour (Suppl. Fig. 3, control Ig vs. rP-sel-Fc groups), as compared with the slow induction of procoagulation activity, which required up to six hours (Suppl. Fig. 2, control Ig vs. rP-sel-Fc groups). This is likely due to anti-inflammation, which can be more easily and immediately induced by the competing interaction of P-sel with its ligands (Fig. 6C); whereas the procoagulant effect requires more time to further elicit TF^+^-MPs (Fig. 6E–G). A period of 6–72 h was required for sP-sel to induce a considerable level of procoagulant TF^+^-MPs *in vivo*^41^. Second, inflammatory mediators can induce thrombosis and procoagulant responses^42^,^43^,^44^,^45^; and anti-inflammatory treatments can also ameliorate hypercoagulation and vaso-occlusion *in vivo*^46^,^47^. This suggests that the first initiated anti-inflammatory effect of sP-sel (Fig. 6B–D, pathway 1) should also, at least in part, participate in the reduction of hypercoagulation burden and preserve functional coagulation machineries, such as platelets and plasma coagulation factors, following the venom challenges. This is in agreement with our data (Fig. 3D,F, venom vs. venom + rP-sel-Fc groups). Third, our TNF-α analysis revealed that snake venom induced the greatest pathological impact within the first hour (Suppl. Fig. 3, 1 h groups); the envenoming mice gradually recovered from the acute inflammatory phase following the second hour after the venom challenges (reduced TNF-α levels; Suppl. Figs 3, 2 and 4 h groups). Therefore, according to the protective effect demonstrated in this study, the late induction of the procoagulant effect of sP-sel involves an intricate regulation that is likely elicited in the last possible moment during the recovery phase for the prevention of hemorrhage and related complications.

Accordingly, our model can also be illustrated in a chronological order, in which pathways 1 and 2 in Fig. 6 are indicated as innate immune and coagulation balance, respectively (Fig. 6; Suppl. Fig. 1A, normal; Suppl. Fig. 1B–D, sP-sel-mediated rebalance from envenomation pathogenesis). Note that here, we highlighted a process in Suppl. Fig. 1B–C, in which the snake venom-induced hypercoagulation is converted to hypocoagulation due to overconsumption of platelets and coagulation factors^1^,^5^,^25^,^48^. This process causes secondary complications, namely hypocoagulation and hemorrhage^1^,^5^,^49^. The procoagulant property of sP-sel is theoretically helpful for rescuing the coagulant defect at this stage. Notably, sP-sel-induced TF^+^-MPs preferentially translocate to the sites of injuries and facilitate thrombi formation^50^,^51^. This is beneficial for rebalancing coagulation hemostasis to stop bleeding specifically at the damaged tissues. Several P-sel blocking agents are currently under clinical trials for managing inflammatory and vascular diseases^27^,^52^,^53^. These agents may be useful to control snake venom-induced complications, as we have demonstrated in the ameliorative effect of anti-P-sel Ig in this study. However, when compared with rP-sel-Fc, these agents do not possess the property for triggering the PSGL-1-mediated procoagulant rescue pathway (Fig. 6, pathway 2); therefore, their rescue efficiency for envenomation is theoretically lower than that of rP-sel-Fc. However, because rP-sel-Fc has procoagulant properties, additional developments involving rP-sel-Fc and any form of sP-sel should be considered with caution when determining the optimal dosage for preventing overdose-induced hypercoagulation. Additionally, whether history of cardiovascular disease influences the treatment outcome of rP-sel-Fc is also a critical categories warranting further investigation.

In summary, our data suggest that the sP-sel-mediated enhancement of hemostasis has therapeutic potential in clinical settings involving deficient coagulation. In contrast to coagulation-suppressive drugs, coagulation-enhancing agents have rarely been developed, likely because of the risk of thrombosis. Consequently, an effective coagulation-enhancing agent suitable for managing internal hemorrhage in the acute phase is still urgently needed. Circulating sP-sel has been considered a marker of various disorders and plays a pathological role in various diseases. Here, we demonstrated that injections of rP-sel-Fc are beneficial for mice in surviving snake venom-challenges, suggesting that an increase in sP-sel is a physiological response of hemostasis. Therefore, as a major component of a hemorrhage-elicited self-rescue response, sP-sel may be useful in the development of a new therapy for managing hemorrhage-related diseases such as venomous snakebites. This may be particularly useful for health care agencies in remote areas that cannot afford to maintain diverse antivenoms.

## Materials and Methods

### Chemicals, antibodies and snake venom

Chemicals and all snake venoms were purchased from Sigma-Aldrich (St. Louis, MO). An anti-P-sel antibody and an isotype control Ig were purchased from BD Pharmingen Taiwan (Taipei, Taiwan) and Enzo Life Sciences (Blossom Biotechnologies, Taipei, Taiwan), respectively. Recombinant P-sel IgG-Fc fusion protein (rP-sel-Fc) was purchased from R&D Systems (Minneapolis, MN). Using previously described immunization methods^54^,^55^,^56^,^57^, polyclonal anti-P-sel Igs were obtained from rP-sel-Fc immunized rabbits for neutralization analysis.

### Mice

The C57BL/6J wild-type mice (males, 8–12 weeks old) were purchased from the National Laboratory Animal Center (NLAC), Taipei, Taiwan. C57BL/6J mice deficient in P-selectin (*Selp*^−/−^; B6.129S-*Selp*^*tm1Bay*^/J)^58^ and P-selectin ligand 1 (PSGL-1) (*Selplg*^−/−^; B6.129-*Selplg*^*tm1Rpmc*^/J) were obtained from the Jackson Laboratory (Bar Harbor, ME). These KO mice were backcrossed with the parental C57BL/6J mouse strain for at least six generations. All mouse strains were housed in the Laboratory Animal Center of Tzu Chi University. At the end of the experiment, the surviving mice were then euthanized with CO_2_ following the National Institutes of Health guideline^59^. In the mortality experiments, the mice were examined every 12 h for up to 7 d. No additional death was observed 7 d after the LT treatments because all surviving mice were monitored every day for 2 mo. Experimental methods in this study were conducted in agreement with National (Taiwan Animal Protection Act, 2008) directive for protection of laboratory animals. All experimental protocols for examining the experimental animals were approved by the Animal Care and Use Committee of Tzu-Chi University, Hualien, Taiwan (approval ID: 103050, 103058).

### Experimental administration

In the mice experiments, reagents were intravenously injected through the retro-orbital venous plexus^60^,^61^ using the following doses: rP-sel-Fc, 1.2 mg/kg^30^; anti-P-sel Ig, 1.2 mg/kg; and isotype control IgG, 1.2 mg/kg. The isotype-matched control IgG was used as a control protein in the *in vivo* experiments because rP-sel is an IgG-Fc fusion protein. Venom doses were using as following: *Crotalus atrox*: sublethal doses 0.2 mg/kg, 1 mg/kg; lethal doses 3 mg/kg, 6 mg/kg. *Crotalus adamanteus*: lethal dose 3 mg/kg. *Crotalus basiliscus*: lethal dose 12 mg/kg. *Agkistrodon contortrix*: lethal dose 4 mg/kg.

### Plasma clotting time analysis

The plasma recalcification clotting time was measured using previously described methods^30^,^59^. Before (0 h) and after (2, 4 and 6 h) the treatments of control Ig and rP-sel-Fc, the blood samples of mice were collected from their retro-orbital venous plexus using plain capillary tubes (Thermo Fisher Scientific Taiwan, Taipei, Taiwan) and collected into polypropylene tubes (Eppendorf; Fisher Scientific) containing anticoagulant acid-citrate-dextrose solution (ACD; 38 mM citric acid, 75 mM sodium citrate, 100 mM dextrose)^8^,^58^,^59^,^62^. Platelet-poor plasma (PPP) was prepared by centrifugation at 1,500 × g for 20 min. PPP was centrifuged once again for 3 min at 15,000 × g to remove contaminating cells from the plasma. Plasma clotting was induced under stirring conditions (800 rpm) at 37 °C in an aggregometer (Model 600B, Ion-Trace, Stouffville, Canada)^63^ by adding a volume of prewarmed 20 mM CaCl_2_ solution to an equal volume of plasma in a siliconized tube. The time (in seconds) needed to clot was determined. The activated partial thromboplastin time (APTT) and prothrombin time (PT) of mice challenged with snake venoms were measured. APTT and PT analyses were performed using a coagulometer (ACL-Futura Plus, Instrumentation Laboratory, Milan, Italy), following the manufacturer’s instructions as described^59^,^64^,^65^.

### Platelet count analysis

Whole blood (50–80 μl) samples of mice were collected from retro-orbital venous plexus and mixed with anticoagulant ACD solution in Eppendorf tubes. Platelet counts were then measured by a hematology analyzer (KX-21N, Sysmex) as described^58^,^60^.

### ELISA and flow cytometry analysis

A standard ELISA protocol was conducted^55^,^66^ using a microplate reader as previously described^67^,^68^,^69^. The circulating levels of sP-sel, IL-1β and TNF-α of experimental mice were determined by mouse sP-Selectin/CD62P, IL-1β and TNF-α ELISA kits (R&D Systems and BioLegend, San Diego, CA)^56^,^69^; calculations were made by comparing the specific sP-sel, IL-1β and TNF-α values with standard curves of titrated P-sel, IL-1β and TNF-α, respectively. Mouse microparticle (MPs) rich plasma was prepared by removing blood cells (including platelets) through centrifugation. To analyze the surface tissue factor (TF) expression of mouse MPs, a flow cytometer (FACScalibur, BD Biosciences, CA) was used^58^,^62^. Fluorescent anti-TF antibody was purchased from Abcam (Cambridge, MA).

### Statistics

The means, standard deviations, and statistics of the experimental data were quantified using software Microsoft Office Excel 2003, SigmaPlot 10, and SPSS 17. The statistical significance of the data was further determined using one-way ANOVA followed by a post hoc Bonferroni-corrected t test. The Kaplan Meier curves are plotted using the Online Application for the Survival Analysis of Lifespan Assay (http://sbi.postech.ac.kr/oasis)^70^. A probability of type 1 error α = 0.05 was determined to be the threshold of statistical significance.

## Additional Information

**How to cite this article**: Sun, D.-S. *et al*. Soluble P-selectin rescues viper venom–induced mortality through anti-inflammatory properties and PSGL-1 pathway-mediated correction of hemostasis. *Sci. Rep.* **6**, 35868; doi: 10.1038/srep35868 (2016).

## Supplementary Material

Supplementary Information

Supplementary Information
