# Supplementary material for: Soluble P-selectin rescues viper venom–induced mortality through anti-inflammatory properties and PSGL-1 pathway-mediated correction of hemostasis
Source: Sci Rep. 2016 Oct 25;6:35868. doi: 10.1038/srep35868 (PMC5078805; doi:10.1038/srep35868)
Supplement: Supplementary Information [file srep35868-s1.doc]

**Supplemental Materials:**

Soluble P-selectin rescues viper venom–induced mortality through anti-inflammatory properties and PSGL-1 pathway-mediated correction of hemostasis

Der-Shan Sun ^1,2^, Pei-Hsun Ho ^2^ and Hsin-Hou Chang ^1,2 *^

^1^ Department of Molecular Biology and Human Genetics, Tzu-Chi University, Hualien 970, Taiwan.

^2^ Center for Vascular Medicine, Tzu-Chi University, Hualien 970, Taiwan.

* Address correspondence to: Hsin-Hou Chang, Department of Molecular Biology and Human Genetics, Tzu-Chi University, Hualien 970, Taiwan.

Phone: 88638565301 ext 2667 ; Fax: 88638578386 ; E-mail: [hhchang@mail.tcu.edu.tw](mailto:hhchang@mail.tcu.edu.tw)

Supplemental figures 1-3.

**Suppl. Fig. 1**


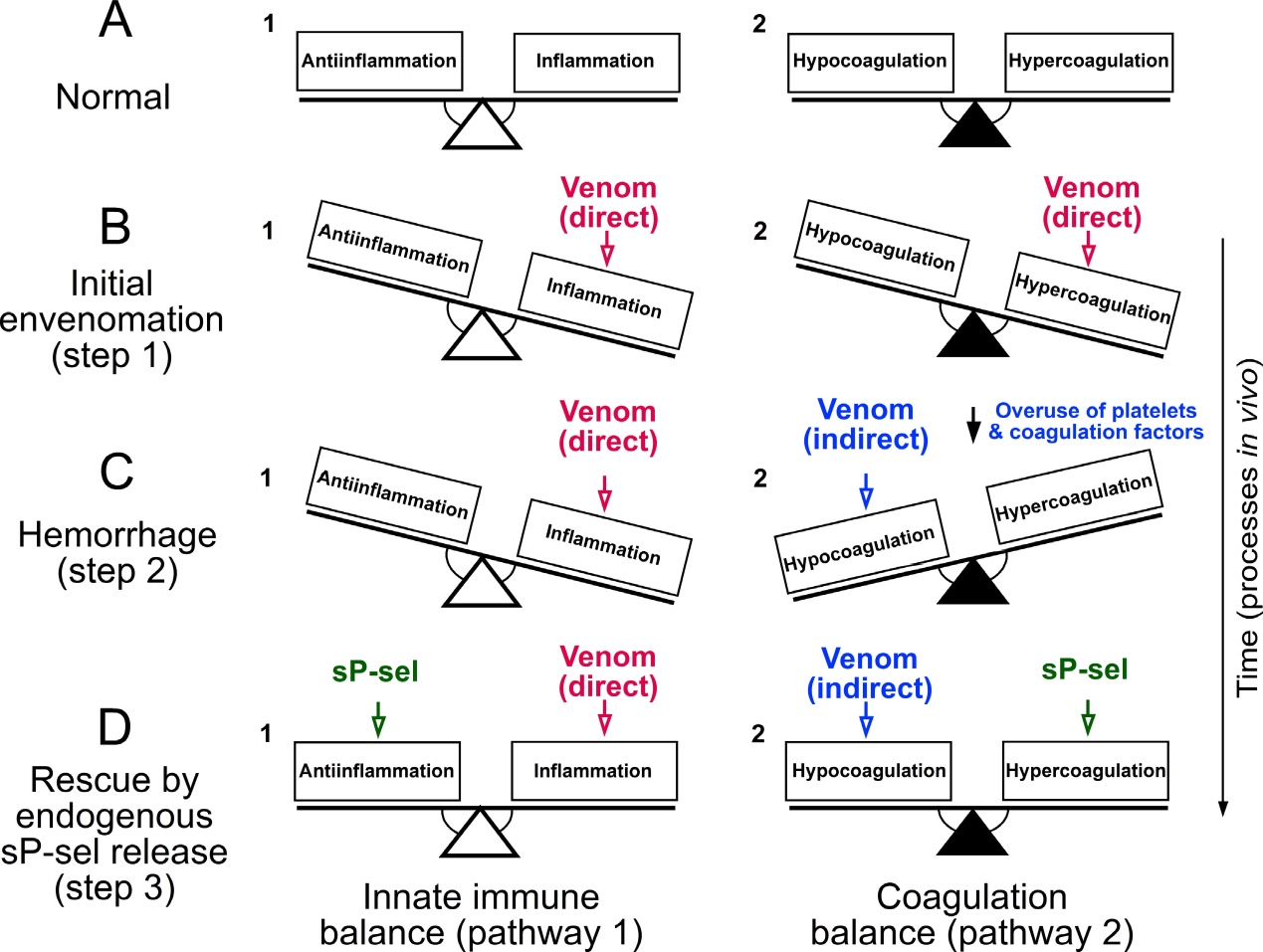


**Suppl. Fig. 1. Hypothetical model of the regulations of innate immune and coagulation homeostasis of sP-sel-mediated rescue after envenomation.** (A) Illustration indicates balanced innate immune (A-1) and coagulation (A-2) homeostasis. (B) After challenged with the snake venom, the balance shifted to enhanced inflammation (B-1) and hypercoagulation (B-2) (red arrows, step 1). The venom directly induced a pathogenic acute coagulant activation (B-2), which results in rapid and high level consumption and exhaustion of platelets and coagulation factors, and indirectly induced a hypocoagulable state (C-2) (blue arrows, step 2). With antiinflammatory and procoagulant properties, the release of endogenous sP-sel rebalanced the innate immune and coagulation systems of the host (D) (green arrows, step 3). The innate immune and coagulation regulations (in steps 1-3, B-D), are equivalent to the pathways 1 and 2 of figure 6, respectively.

**Suppl. Fig. 2**


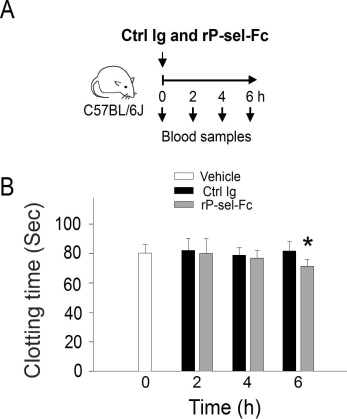


**Suppl. Fig. 2.** **Compared to the antiinflammatory property, recombinant P-sel-Fc induced a relatively slow procoagulant response 6 hour after the treatments.** Experiment outline (A), and the mouse recalcification plasma clotting time before (0 h) and after (2, 4, 6 h) the control Ig (Ctrl Ig) and recombinant P-selectin IgG-Fc fusion protein (rP-sel-Fc) treatments (B), are indicated. n = 6 (B; 3 independent experiments with 2 replicates). * *P* < 0.05, vs. respective control Ig treated groups (B). The mouse drawing used in this figure was originally published in the Blood journal: Huang, H. S., Sun, D. S., Lien, T. S. and Chang, H. H. Dendritic cells modulate platelet activity in IVIg-mediated amelioration of ITP in mice. Blood, 2010; 116: 5002–5009. © the American Society of Hematology.

**Suppl. Fig. 3**


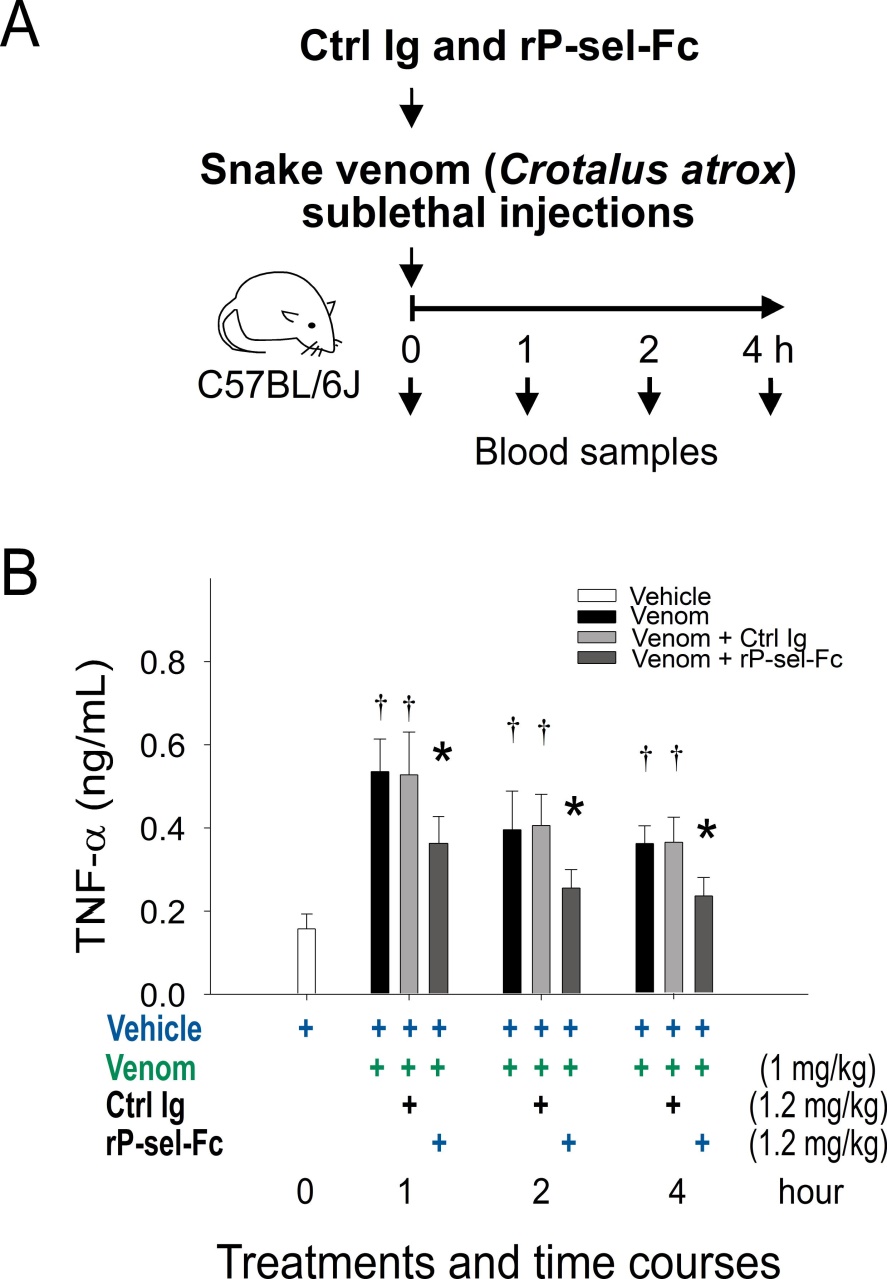


**Suppl. Fig. 3.** **Compared to the procoagulant property, recombinant P-sel-Fc induced a relatively fast antiinflammatory response within 1 hour after the treatments.** Experiment outline (A), and the mouse circulating TNF-α levels before (0 h) and after (1, 2 and 4 h) the snake venom challenges, with or without additional control Ig (Ctrl Ig) and recombinant P-selectin IgG-Fc fusion protein (rP-sel-Fc) treatments (B), are indicated. n = 6 (B; 3 independent experiments with 2 replicates). † *P* < 0.05, vs. the vehicle group, * *P* < 0.05, vs. respective venom + control Ig groups (B). The mouse drawing used in this figure was originally published in the Blood journal: Huang, H. S., Sun, D. S., Lien, T. S. and Chang, H. H. Dendritic cells modulate platelet activity in IVIg-mediated amelioration of ITP in mice. Blood, 2010; 116: 5002–5009. © the American Society of Hematology.
